# Radio-anatomical evaluation of clinical and radiomic profile of multi-parametric magnetic resonance imaging of de novo glioblastoma multiforme

**DOI:** 10.1186/s43046-024-00217-3

**Published:** 2024-04-22

**Authors:** H. Shafeeq Ahmed, Trupti Devaraj, Maanini Singhvi, T. Arul Dasan, Priya Ranganath

**Affiliations:** 1 Department of Radio-Diagnosis, Bangalore Medical College and Research Institute, Bangalore, 560002 India; 2 Department of Anatomy, Bangalore Medical College and Research Institute, Karnataka Bangalore, 560002 India

**Keywords:** Glioblastoma, Neurology, Radiology, MRI, Neuro-oncology

## Abstract

**Background:**

Glioblastoma (GBM) is a fatal, fast-growing, and aggressive brain tumor arising from glial cells or their progenitors. It is a primary malignancy with a poor prognosis. The current study aims at evaluating the neuroradiological parameters of de novo GBM by analyzing the brain multi-parametric magnetic resonance imaging (mpMRI) scans acquired from a publicly available database analysis of the scans.

**Methods:**

The dataset used was the mpMRI scans for de novo glioblastoma (GBM) patients from the University of Pennsylvania Health System, called the UPENN-GBM dataset. This was a collection from The Cancer Imaging Archive (TCIA), a part of the National Cancer Institute. The MRIs were reviewed by a single diagnostic radiologist, and the tumor parameters were recorded, wherein all recorded data was corroborated with the clinical findings.

**Results:**

The study included a total of 58 subjects who were predominantly male (male:female ratio of 1.07:1). The mean age with SD was 58.49 (11.39) years. Mean survival days with SD were 347 (416.21) days. The left parietal lobe was the most commonly found tumor location with 11 (18.96%) patients. The mean intensity for T1, T2, and FLAIR with SD was 1.45E + 02 (20.42), 1.11E + 02 (17.61), and 141.64 (30.67), respectively (*p* =  < 0.001). The tumor dimensions of anteroposterior, transverse, and craniocaudal gave a z-score (significance level = 0.05) of − 2.53 (*p* = 0.01), − 3.89 (*p* < 0.001), and 1.53 (*p* = 0.12), respectively.

**Conclusion:**

The current study takes a third-party database and reduces physician bias from interfering with study findings. Further prospective and retrospective studies are needed to provide conclusive data.

## Introduction

Glioblastoma (GBM) is a highly aggressive and lethal brain tumor that originates from glial cells or their progenitors. It is considered a primary malignancy with a grim prognosis. While GBM infiltrates surrounding brain tissue, it does not metastasize to distant organs [1]. In 1940, Hans Joachim Sherer, a German pathologist, coined the terms “primary” and “secondary” GBM in Antwerp [2]. Histologically, primary and secondary GBMs are similar, but they differ in their genetic profiles. Primary GBMs typically develop in older patients and commonly exhibit epidermal growth factor receptor (EGFR) overexpression, mutations in PTEN (MMAC1), deletions in CDKN2A (p16), and, occasionally, amplification of MDM2. On the other hand, secondary glioblastomas occur in younger patients and often involve TP53 mutations. Primary or de novo GBM constitutes approximately 80% of all GBMs [3].

GBMs are classified as stage-4 tumors and can originate in the brain as primary tumors or evolve from lower-grade astrocytomas. In adults, GBMs commonly occur in the frontal and temporal lobes of the brain. The exact causes or risk factors for developing GBMs are not yet fully understood. However, no specific risk factors have been identified thus far. It is crucial to note that if left untreated, GBMs have a devastating prognosis, often leading to death within 6 months or even less [4, 5]. The migration of malignant cells into the surrounding brain tissue, the occurrence of seizures, increased intracranial pressure, resistance to conventional therapies, and the limited regenerative capacity of neurons all contribute to the high fatality rate of GBMs [1]. It is noteworthy that mutations in the isocitrate dehydrogenase-1 (IDH1) and IDH2 genes are commonly observed in 70–80% of low-grade gliomas and secondary GBMs but only in 5–10% of de novo GBMs. These genetic variations provide important insights into the distinct molecular characteristics and clinical behavior of different subtypes of GBMs.

The average annual age-adjusted incidence rate (IR) of GBM in the United States is 3.19 per 100,000 persons. However, this rate is 2.5 times higher for African Americans and European Americans. Novel drugs in the context of GBM including vinorelbine and gemcitabine have been studied due to rising incidence. GBMs account for 54% of all gliomas and 16% of all primary central nervous system (CNS) tumors. Despite surgical interventions, chemotherapy, and radiotherapy, the median survival for GBM patients remains less than 14 months [5]. In light of this, the current study aims to evaluate the neuroradiological parameters of de novo GBM by analyzing brain MRI scans obtained from a publicly available database. This analysis will provide valuable insights into the imaging characteristics and features of GBMs, contributing to a better understanding of this aggressive brain tumor.

## Methods

### Data source

The current study utilizes brain MRI scans of de novo GBM patients obtained from The Cancer Imaging Archive (TCIA), which is a part of the National Cancer Institute [6]. Specifically, the dataset used is the multi-parametric magnetic resonance imaging (mpMRI) scans for de novo GBM patients from the University of Pennsylvania Health System, known as the UPENN-GBM dataset [7]. This dataset consists of mpMRI images captured during routine clinical radiologic exams at the preoperative baseline time-point. By analyzing these MRI scans, the study aims to examine the neuroradiological parameters and characteristics of de novo GBM, providing valuable insights into the imaging features of this aggressive brain tumor.

### Sociodemographic parameters

All MRI scans were supplemented with corresponding patient sociodemographic data and clinical outcomes (e.g., overall survival, genomic information) at the time of admission. Additionally, molecular analysis was conducted to determine the IDH-1 mutation status using next-generation sequencing (NGS) and/or immunohistochemistry for IDH1-R132H. Furthermore, the methylation status of O(6)-methylguanine-DNA methyltransferase (MGMT) was also assessed. [8–10] These molecular parameters provide important the genetic profile and potential prognostic markers of the de novo GBM patients in the study.

### Imaging parameters

To enhance the analysis of the MRI scans, computer-aided segmentation labels were generated for various histologically distinct subregions of the tumor. These segmentation labels were further manually corrected to ensure accuracy. Moreover, the entire brain was segmented and co-registered with the corresponding mpMRI volumes. This comprehensive approach provided a rich panel of radiomic parameters that were co-registered with the segmented images in NIfTI format. Prior to segmentation, the scans underwent skull stripping and co-registration. The tumor segmentation labels were then generated using an automated computational method. This integration of computer-aided segmentation and radiomic analysis enables a detailed evaluation of the tumor subregions and their radiomic features, contributing to a more comprehensive understanding of the de novo GBM.

A stringent set of inclusion and exclusion criteria was employed to ensure the selection of eligible patients for the study. Patients with unknown days of survival and those lacking both sagittal and axial sections in their MRI scans were excluded from the analysis. Following the application of these predefined exclusion criteria, the initial pool of 630 patients was narrowed down to a final cohort of 58 individuals, who met all the necessary criteria for comprehensive evaluation and analysis.

### Image analysis

A diagnostic radiologist with expertise in neuroimaging reviewed all the MRIs included in the study. The radiologist meticulously analyzed the images and recorded important tumor parameters, including the anteroposterior (AP), transverse (TR), and craniocaudal (CC) lengths, utilizing both the axial and sagittal sections. The location of the tumor within the brain was also carefully noted. As CT scans were not available for evaluation, the analysis focused exclusively on the MRI images. The radiological findings were then cross-referenced and validated with relevant clinical data extracted from the dataset, ensuring a comprehensive and robust assessment of the neuroimaging features.

### Statistical analysis

The collected data was meticulously tabulated using Microsoft Excel and organized in a clear and concise manner. Descriptive statistics were used to summarize the data, with continuous variables presented as mean with standard deviation (SD) and categorical variables reported as number (n) with percentage (%). To assess the statistical significance of differences between numerical values in qualitatively separated data, a repeated measure ANOVA test was conducted. Pearson correlation analysis was performed to determine correlation coefficients between different numerical datasets. Additionally, a two-tailed single-sample z-score was calculated to evaluate the variance within the dataset and ensure a normal distribution of the data. Visual representations of the data were generated using box plots, scatterplots, and raincloud plots to facilitate comparisons between numerical and qualitative data points. The threshold for statistical significance was set at *p* < 0.05.

## Results

The current study enrolled a total of 58 subjects, with a majority being male (51.72%). The mean age of the subjects was 58.49 years with a SD of 11.39 years. The sociodemographic characteristics of the subjects are presented in Table 1. The mean survival duration was 347 days with a standard deviation of 416.21 days. Genetic typing revealed that the most common genetic profile was wild type, observed in 40 subjects (68.9%), followed by not otherwise specified/not elsewhere classified (NOS/NEC) in 16 subjects (27.58%). A correlation analysis between age and survival days showed a correlation coefficient (r) of − 0.038 (*p* = 0.009), indicating a weak negative correlation between these variables.

**Table 1:** Sociodemographics of the patients

**Sociodemographic parameters**	**Results** *N with % or mean with SD*
**Mean age in years with SD**	58.49 (11.39)
**Sex**	
Male	30 (51.72%)
Female	28 (48.28%)
**Mean survival days with SD**	347 (416.21)
**Genetic typing**	
Wild type	40 (68.9%)
NOS/NEC	16 (27.58%)
Mutated	2 (3.44%)

**Fig. 1:**
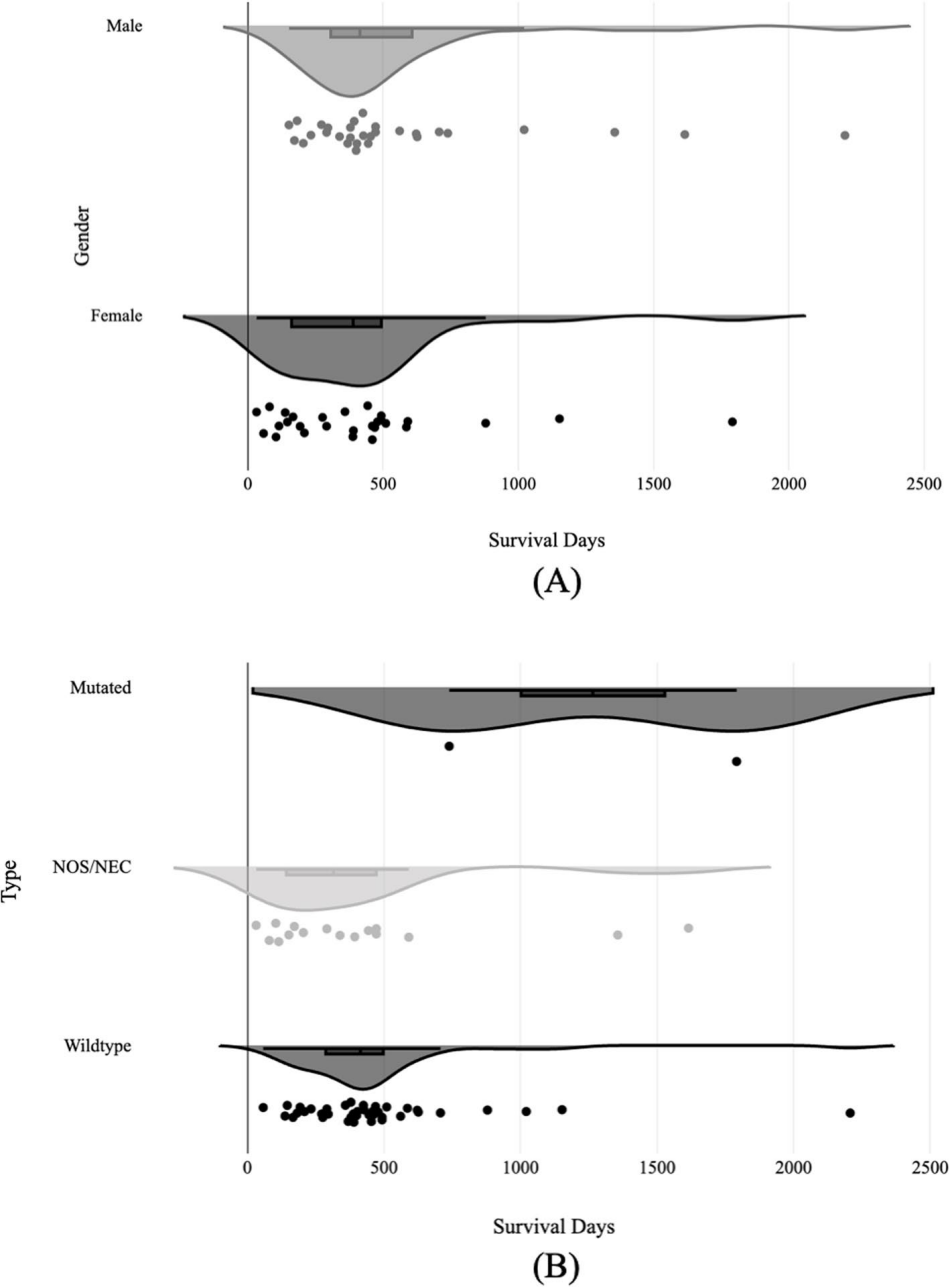
Raincloud plot observing variation between survival days for **A** males and females and **B** between the different variants

Figure 1 presents a raincloud plot illustrating the variation in survival days for (A) males and females and (B) different genetic variants. Among males, the mean survival duration was 553.8 days with a SD of 453.15 days, while among females it was 419.71 days with an SD of 368.13 days (*p* = 0.152). Comparing the genetic variants, individuals with the mutated-type variant had a longer mean survival duration of 1265 days with an SD of 743.87 days, compared to 427 days with an SD of 447.28 days and 475.1 days with an SD of 359.72 days for the wild-type and NOS/NEC variants, respectively (*p* = 0.02).

Table 2 presents the radiological parameters of the study cohort. The most frequently observed tumor location was the left parietal lobe, with 11 (18.96%) patients. This was followed by the right frontal lobe and left temporal lobe, each observed in 9 (15.51%) patients. The mean dimensions of the tumor, measured as AP, TR, and CC lengths with SD, were 33.95 (10.91), 25.18 (10.27), and 30.96 (10.5) pixels, respectively.

**Table 2:** Radiological parameters of patients

**Radiological parameters**	**Results** *N with % or mean with SD*
**Tumor locations**	
Diencephalon	1 (1.72%)
Left external capsule	1 (1.72%)
Left frontal lobe	4 (6.89%)
Left occipital lobe	1 (1.72%)
Left parietal lobe	**11 (18.96%)**
Left temporal lobe	**9 (15.51%)**
Left parietal lobe crossing the midline	1 (1.72%)
Left parietal lobe and left occipital lobe	2 (3.44%)
Left temporal lobe crossing the midline	1 (1.72%)
Right capsuloganglionic region	1 (1.72%)
Right frontal lobe	**9 (15.51%)**
Right parietal lobe	7 (12.06%)
Right temporal lobe	6 (10.34%)
Right external capsules, insular ribbon, and temporal lobe	1 (1.72%)
Right frontal lobe and right temporal lobe	2 (3.44%)
Right thalamus, cerebral peduncle, and mid brain	1 (1.72%)
**Tumor dimensions (pixels)**	
Anteroposterior	33.95 (10.91)
Transverse	25.18 (10.27)
Craniocaudal	30.96 (10.5)

**Fig. 2:**
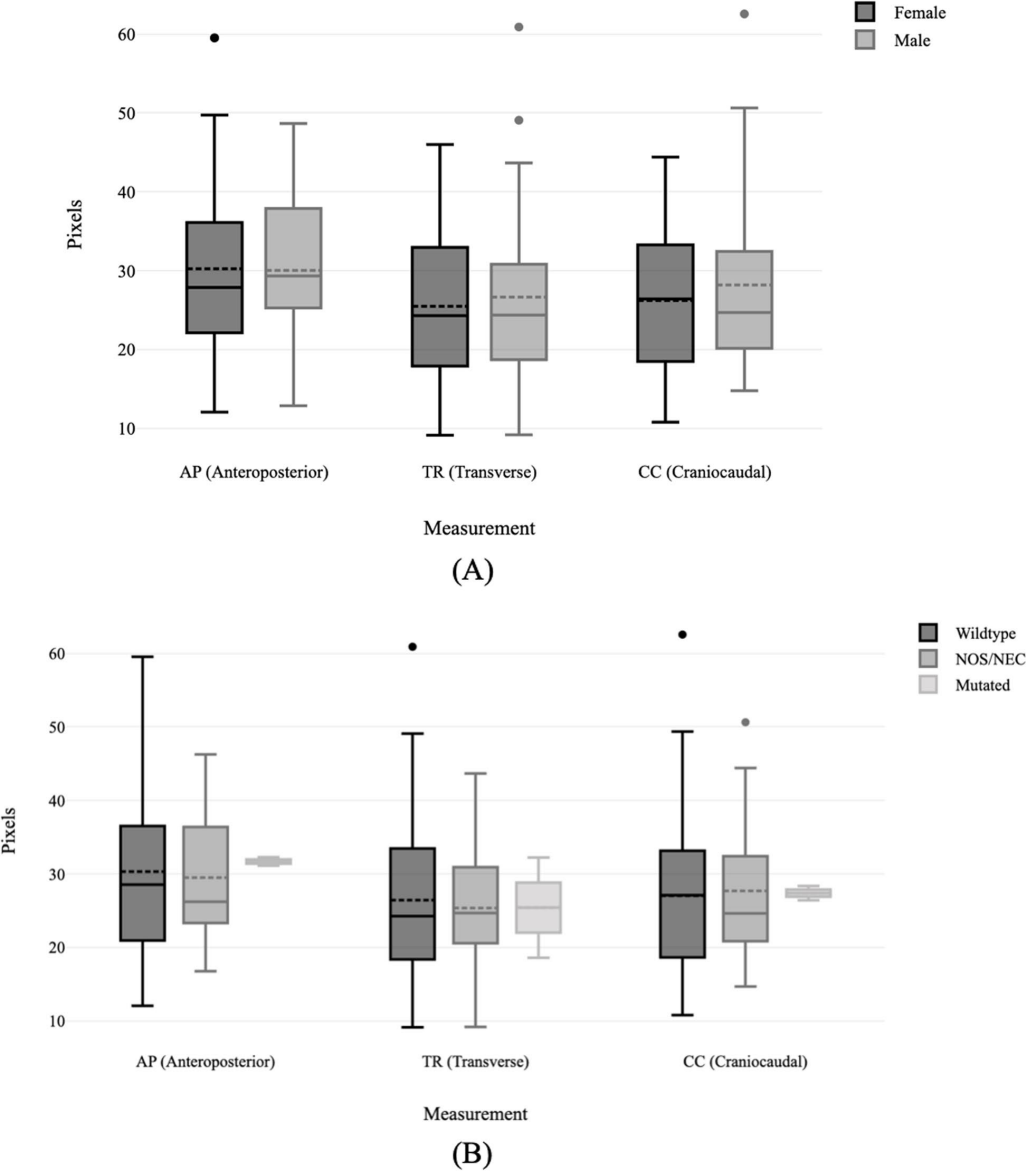
Box plot observing variation between AP, TR, and CC pixels for **A** males and females and **B** between the different variants

Figure 2 illustrates the variations in the AP, TR, and CC measurements based on (A) gender and (B) different variants. In terms of gender, the box plot reveals that female subjects had a higher mean AP length of 30.25 pixels compared to males. For TR length, males had a higher mean of 26.66 pixels. Similarly, males also exhibited a higher mean CC length of 28.19 pixels.

**Fig. 3:**
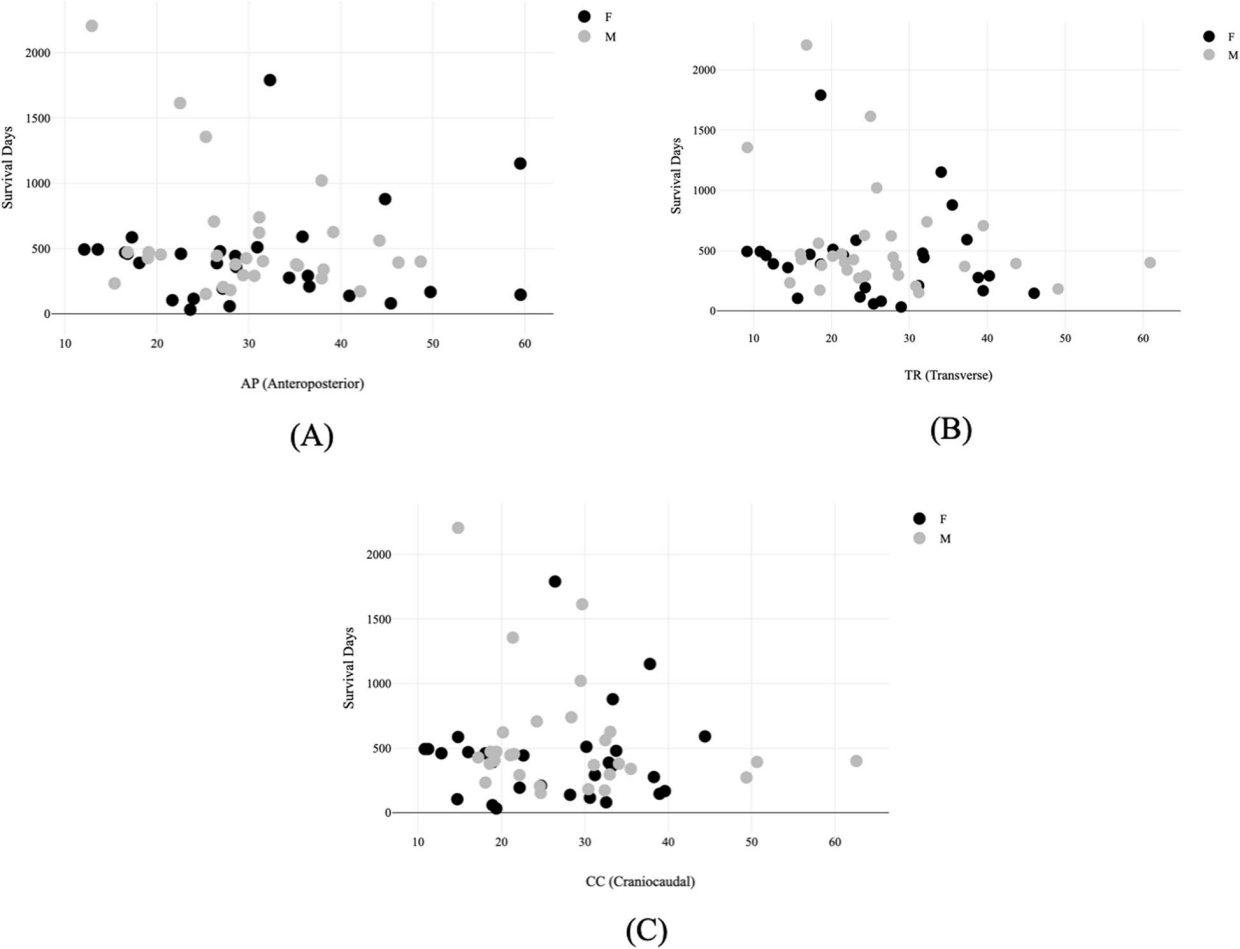
Scatterplot observing variation between survival days depending on **A** AP, **B** TR, and **C** CC pixel size

Figure 3 depicts a scatterplot graph showing variations in survival days depending on different (A) AP, (B) TR, and (C) CC for males and females.

Table 3 presents the radiomic data of the subjects, including various parameters obtained from T1-weighted scans, T2-weighted scans, and FLAIR MRI scans. The mean intensity values for T1, T2, and FLAIR were 1.45E + 02 (20.42), 1.11E + 02 (17.61), and 141.64 (30.67), respectively, demonstrating statistically significant differences (*p* =  < 0.001). Additionally, other parameters such as skewness, range, maximum, and minimum were also recorded. Among these parameters, FLAIR exhibited the highest maximum energy at 234.71 (26.78), while T1 had the highest median intensity of 144.62 (21.5).

**Table 3:** Radiomics of different MRI protocols

**Radiomic features (intensity)**	**T1**		**T2**		**FLAIR**		***p*-value**
	Mean	SD	Mean	SD	Mean	SD	
Coefficient of variation	0.12	0.03	0.21	0.04	0.19	0.05	< 0.001
Energy	1,157,082,922	883,306,960.40	817,544,941.90	705,996,957.90	1,318,522,850.00	1,117,225,065.00	0.012
Interquartile range	24.17	4.33	33.16	10.16	36.26	12.24	< 0.001
Kurtosis	4.09E + 00	1.97	3.39	1.55	3.52	1.88	0.085
Maximum	1.45E + 02	23.36	215.67	29.34	234.71	26.78	< 0.001
Mean	1.45E + 02	20.42	1.11E + 02	17.61	141.64	30.67	< 0.001
Mean absolute deviation	− 9.4E-13	0.00	3.01E-13	0	8.13E-13	0	0.05
Median	144.62	21.50	110.24	18.65	141.69	32.86	< 0.001
Median absolute deviation	0.42	2.42	0.34	3.08	− 0.05	3.93	0.69
Minimum	46.84	35.19	24.98	20.10	31.28	28.43	< 0.001
Mode	140.03	29.57	110.83	22.42	142.21	44.59	< 0.001
Ninetieth percentile	168.78	20.57	141.33	24.36	174.36	34.12	< 0.001
Range	170.29	39.90	190.69	35.76	0.13	0.04	< 0.001
Skewness	− 0.03	0.67	0.24	0.47	203.43	39.10	0.05
Standard deviation	18.52	3.68	23.73	5.83	144.09	30.56	< 0.001

**Fig. 4:**
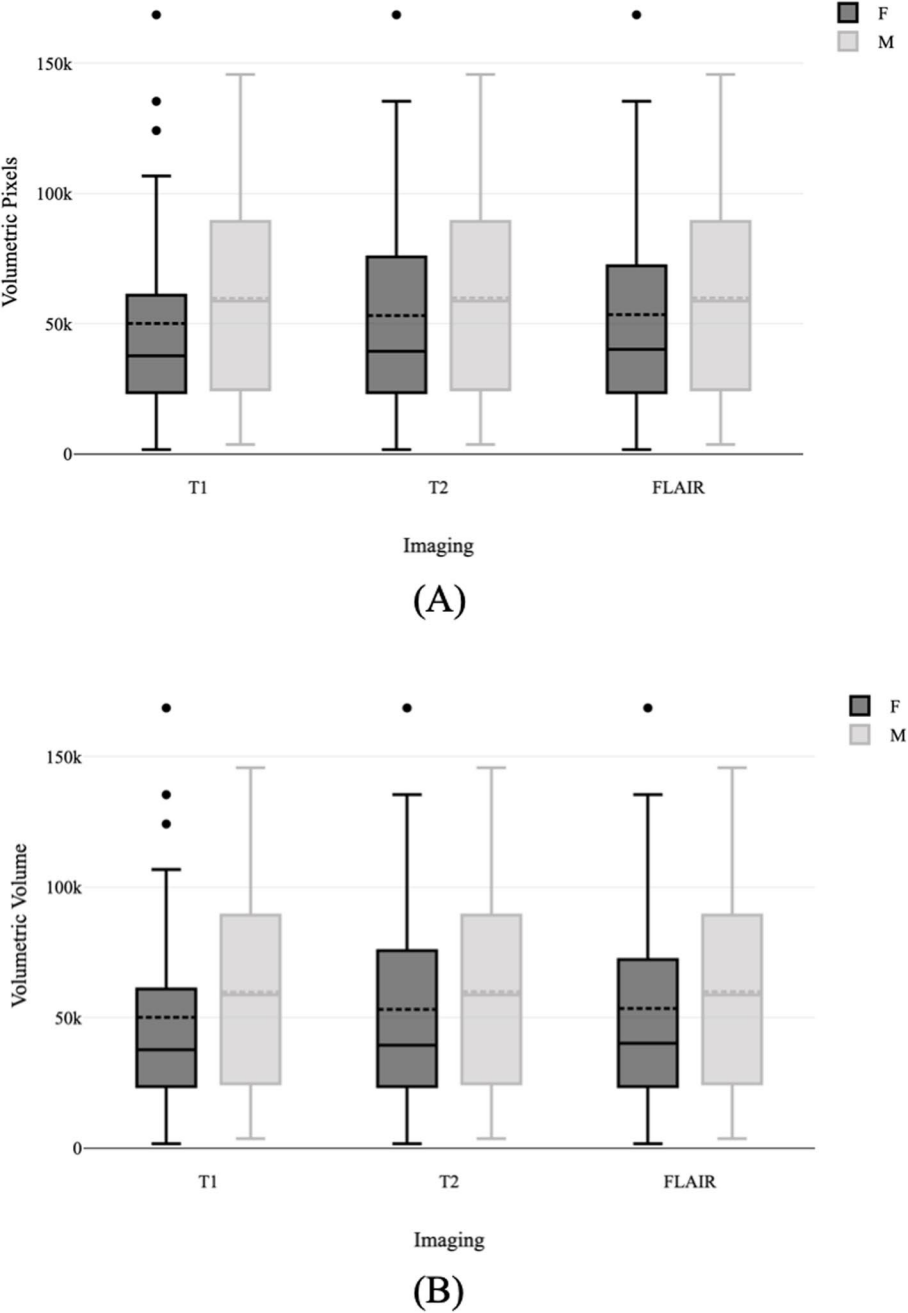
Box plot observing variation between **A** volumetric pixels and **B** volumetric volume for T1, T2, and FLAIR imaging parameters

Figure 4 shows a box plot observing variation between (A) volumetric pixels and (B) volumetric volume for T1, T2, and FLAIR imaging parameters. Males have a higher mean than females in all three imaging parameters in both volumetric pixels and volume.

## Discussion

The current study represents a pioneering effort in utilizing an internationally accessible open-access database to investigate the radiological profile of de novo GBM. Due to the relatively low incidence of this disease compared to other metastatic cancers, comprehensive discussions on the subject pose challenges. The study population exhibited a slight male predominance, with a male-to-female ratio of 1.07:1, aligning with existing data from the United States [11]. The mean survival duration, regardless of the treatment protocol employed, was 347 (416.21) days, while the mean age of the subjects was 58.49 (11.39) years. The importance of evaluating the quality of life in these patients and establishing correlations with prognostic factors is underscored by the duration of survival [12, 13]. The majority (68.9%) of patients demonstrated wild-type IDH1 mutations, followed by cases categorized as NOS/NEC.

The mean survival days with SD for males and females were 553.8 (453.15) and 419.71 (368.13) days, respectively. Similarly, the mean survival days with SD for different GBM types were 475.1 (359.72) days for wild type, 427 (447.28) days for NOS/NEC, and 1265 (743.87) days for mutated. The raincloud plot in Fig. 1 provides a visual representation of these differences. It is worth noting that males tended to have a higher number of survival days compared to females, while patients with the mutated type had significantly higher survival days compared to those with wild type and NOS/NEC. However, it is important to consider that the mutated type comprised only two patients, with survival days of 1791 and 739 days, respectively. In a study conducted by Baid et al., a neural network was developed using MRI images, age, and tumor resection status to predict the survival days of GBM patients. The network achieved an accuracy of 70.2% in the training subset and 62.5% and 63.6% in the validation and testing subsets, respectively, with an overall accuracy of 73% for the entire dataset [14]. 

GBMs are typically found in the supratentorial region of the brain, which includes the frontal, temporal, parietal, and occipital lobes. Among these regions, the frontal lobe has the highest incidence of GBMs, followed by the temporal and parietal lobes, with tumors often overlapping multiple lobes. In the current study, the parietal lobe was the most commonly affected region, observed in 21 (36.2%) patients [15]. The dimensions of the tumor, including the anteroposterior (AP), transverse (TR), and craniocaudal (CC) measurements, were analyzed using z-scores to assess their significance. The z-score for AP was − 2.53 (*p* = 0.01), indicating a statistically significant difference from the mean. Similarly, the z-score for TR was − 3.89 (*p* < 0.001), indicating a highly significant difference. However, the CC dimension had a z-score of 1.53 (*p* = 0.12), indicating that the difference was not statistically significant at the specified significance level of 0.05.

Radiomics analysis of multi-parametric MRIs provides valuable information for radiologists in distinguishing between tumor progression and pseudoprogression. Ismail et al. achieved an impressive accuracy of 90.2% in this differentiation by combining T1 and T2/FLAIR images to create a 3D image for surface radiomics extraction [16]. They identified key factors such as the total curvature of the enhancing lesion and the curvature of the peritumoral hyperintense regions from T2/FLAIR images that aided in this distinction. The use of radiomics can significantly reduce the need for invasive biopsies as they contribute to the diagnosis and evaluation of treatment efficacy in patients. It is important to note that approximately 40% of glioblastomas do not respond to chemoradiotherapy and exhibit progression over 6–9 months [17]. Hypoxia, a critical pathway in neovascularization within GBM tumors, contributes to tumor resistance and leads to a poorer prognosis [18]. Beig et al. demonstrated that radiomics analysis can assess the extent of neovascularization and hypoxia before treatment, providing insights into survival prediction [19]. Figure 4 illustrates the impact of volumetric pixels and volumetric volume, two radiomic parameters, on survivability between males and females. These findings underscore the significance of considering different imaging protocols, including T1, T2, and FLAIR, when evaluating and analyzing radiomics in GBM patients.

Prasanna et al. conducted a study to assess the correlation between survivability and mass effect-induced deformation heterogeneity (MEDH) in glioma patients [20]. They found that the expression of MEDH in highly expressed multi-sequence MRIs had a significant impact on survivability, particularly when observed in areas associated with emotion, language comprehension, visual perception, social cognition, somatosensory, motor, and cognitive functions. This highlights the importance of considering the effects of tumor-induced deformation on various cognitive functions when evaluating prognosis in glioma patients. In addition, a review by Boele et al. delves into the psychological challenges faced by patients diagnosed with gliomas. The authors emphasize the impact of psychiatric disturbances, such as mood disorders and cognitive impairments, on patients’ quality of life. Effective management of behavioral changes and psychological symptoms related to tumors in cognitive regions is crucial and often requires appropriate psychotherapy interventions [21]. Chambers et al. have published research focusing on the implementation of psychotherapeutic approaches specifically tailored to address these challenges and improve the well-being of glioma patients [22]. Taken together, these studies highlight the significance of considering the impact of tumor-induced deformations and psychological factors on the prognosis and management of glioma patients. By comprehending and addressing these aspects, healthcare professionals can deliver holistic care that considers both the physical and psychological dimensions of the disease. These studies showcase the importance of assessing these factors within an Asian context, including regions such as the Middle East (encompassing Egypt, Saudi Arabia, and Turkey) and Asian countries like India, Pakistan, and China.

The current study has several limitations that should be acknowledged. Firstly, the study utilized a limited sample size, although it was larger compared to previous studies in the field. This limited sample size may affect the generalizability of the findings to the larger population. Additionally, the study did not correlate the radiological findings with histopathological or genetic findings, which could provide valuable insights into the underlying mechanisms of GBM. Although all patients were diagnosed with GBM based on histopathological assessment, the availability of data regarding specific histopathological and genetic characteristics was limited and not considered in the analysis. Another limitation is the lack of standardization in radiological protocols, including segmentation and image acquisition processes. This may lead to variations in the interpretation of radiological findings and limit the comparability of results across different studies or centers. It is important to consider these variations when interpreting the findings and applying them to real-world clinical scenarios. Despite these limitations, the study has its merits. The utilization of a third-party dataset helps minimize possible biases and enhances the generalizability of the study findings. By utilizing a diverse dataset, the study findings may provide valuable insights into the radiological profile of de novo GBM in real-world clinical settings. However, future studies with larger sample sizes, standardized protocols, and correlations with histopathological and genetic data would further strengthen the understanding of GBM and its radiological characteristics.

## Conclusion

While interpreting our research findings, it is important to consider the limitations discussed earlier. However, the application of radiomics in the context of neuro-oncology offers a fresh perspective and adds to our understanding of the field. Our findings, along with those of other researchers, highlight the significance of radiomics in the clinical assessment of survival outcomes in GBMs and other brain tumors. To further validate and expand upon these findings, future studies should include larger cohorts of patients with complete data availability. Prospective and retrospective studies conducted on such comprehensive datasets would serve as valuable tools in unraveling the true potential and importance of radiomics in predicting prognosis in GBMs.

## Data Availability

The dataset analyzed during the current study are available in the Cancer Imaging Archive repository: https://wiki.cancerimagingarchive.net/pages/viewpage.action?pageId=70225642.

## Abbreviations

GBM: Glioblastoma
EGFR: Epidermal growth factor receptor
IDH: Isocitrate dehydrogenase
IR: Incidence rate
CNS: Central nervous system
TCIA: The Cancer Imaging Archive
mpMRI: Multi-parametric magnetic resonance imaging
NGS: Next-generation sequencing
MGMT: Methylguanine-DNA methyltransferase
AP: Anteroposterior
TR: Transverse
CC: Craniocaudal
SD: Standard deviation
NOS/NEC: Not otherwise specified/not elsewhere classified
MEDH: Mass effect-induced deformation heterogeneity
